# Technical Efficiency of Mexico’s Public Health System in the Delivery of Obstetric Care, during 2012–2018

**DOI:** 10.3390/healthcare12060653

**Published:** 2024-03-14

**Authors:** Belkis Aracena-Genao, René Leyva-Flores, Rene Santos-Luna, Saul Lara-Diaz, Angel Argenis Mejía-Avilez

**Affiliations:** 1 Center for Research in Nutrition and Health (CINyS), National Institute of Public Health (INSP), Cuernavaca 62100, Morelos, Mexico; belkis.aracena@insp.mx; 2 Center for Research in Health Systems (CISS), National Institute of Public Health (INSP), Cuernavaca 62100, Morelos, Mexico; rene.leyva@insp.mx; 3 Center for Research in Evaluation and Surveys, National Institute of Public Health (INSP), Cuernavaca 62100, Morelos, Mexico; 4 Information Center for Public Health Decisions, National Institute of Public Health (INSP), Cuernavaca 62100, Morelos, Mexico; saul@insp.mx (S.L.-D.); ingeniero6@insp.mx (A.A.M.-A.)

**Keywords:** efficiency, delivery of health care, obstetrics, data analysis, Mexico

## Abstract

The objective of this study was to evaluate the technical efficiency of Mexico’s public health system in the delivery of obstetric care from 2012 to 2018. A multi-stage quantitative study of the public health institutions responsible for 95% of the system’s obstetric services was conducted using data envelopment analysis. The efficiency of state-level productive units (decision-making units, or DMUs) was calculated and juxtaposed with the DMUs’ maximum (0.82) and minimum (0.22) scores. Using the outcomes of the initial stage, the average technical efficiency of each institution at the national level was estimated and compared. The results were also utilized to estimate and compare the average efficiency of each state-level health system based on economic characteristics (state GDP per capita). Outputs included prenatal visits and deliveries, while inputs comprised gynecologists, exam rooms, and delivery rooms. Institutional efficiency ranged from 0.16 to 0.82, with an average of 0.417. The Ministry of Health (0.82) and the Mexican Social Security Institute (0.747) exhibited the highest efficiency scores, while the remaining institutions (Institute for Social Security and Services for State Workers [ISSSTE]; Mexican Petroleum [PEMEX]; the Secretary of National Defense [SEDENA]; and the Navy [SEMAR]) scored below the health system average. Of the 153 DMUs, 20% surpassed the maximum (0.82) and 40.6% fell below the minimum (0.22). These findings indicate that 80% of DMUs have unused operational capacity that could be utilized to enhance technical efficiency. No relationship was found between efficiency and the GDP of Mexico’s 32 politico-administrative divisions. The efficiency gap between institutions (0.66) shows that while some DMUs are saturated (exhibiting high efficiency scores), the majority have unused operational capacity. Leveraging this untapped capacity could address the needs of vulnerable populations facing restricted access due to health system fragmentation.

## 1. Introduction

Globally, particularly in developing countries, the provision of maternal health services faces significant hurdles in ensuring both adequate quality and quantity. These challenges result in restricted access to care, and an increased prevalence of risks, complications, and deaths [1,2,3]. Despite the implementation of various policies and strategies to universalize obstetric care delivery [4,5], outcomes remain limited. Alarmingly, considerable proportion of births, mostly among indigenous women, occur without the presence of trained health personnel, causing hundreds of women to die every day. Most of these deaths are concentrated in developing regions, where the maternal mortality ratio was about 14 times higher than in developed regions in 2013 [6].

Insufficient service production is frequently attributed to resource scarcity, which is why policy makers and civil society advocate for higher allocations [7,8,9]. Coupled with resource scarcity are efficiency problems, which further limit the productive capacity of institutions and hinder the delivery of needed services [10].

Efficiency, understood as the relationship between inputs in the production process and the resulting goods and services [11], is achieved when production is either maximized, or the mix of inputs required to produce a specific quantity of outputs is minimized. This definition assumes a scheme in which inputs are used correctly, are working at full capacity, and all involved parties cooperate effectively [10].

In Latin America, health systems are predominantly characterized by fragmentation across various institutions and a segmentation of the population based on labor and economic profiles [12,13]. Interaction between these institutions, if any, is generally limited. In addition to these schemes (which obstruct efficiency), socioeconomic differences between and within countries can influence the performance of the health system’s constituent institutions. Therefore, it is reasonable to expect that countries with superior socioeconomic conditions would have more efficient health systems [14].

In Mexico, classified as a upper-middle-income economy (USD 4466 to USD 13,845) [15], the GDP per capita was USD 9946 in 2019 [16]. Its governmental structure comprises three tiers: a centralized federal level, local state governments, and municipal administrations [17]. Additionally, its health system mirrors the fragmented and segmented organization observed in other Latin American countries [18]. Since its inception in the 1940s, the Mexican health system has been structured to allocate funds by population segments, and since then, it has undergone few structural changes. The federal government established public institutions to directly deliver health services, which can be broadly categorized into two groups. The first category consists of social security institutions, responsible not only for health services but also other functions such as pensions, retirement benefits, housing financing, recreational services, and more. These services are funded through contributions from employers, employees, and the federal government, covering employees and their families, retirees, and pension recipients. The second category comprises social welfare institutions which are exclusively focused on providing health services and funded entirely through general tax revenues. These institutions cover workers in the informal sector, farmers, the unemployed, international migrants, and the families of each of these groups. Private medical institutions offer varying levels of care based on market demand and are financed predominantly through out-of-pocket payments and, to some extent, by health insurance [19,20].

In Mexico, a multitude of public and private institutions operate in a fragmented manner with limited collaboration mechanisms. Notable among these are the social security institutions, including the Mexican Social Security Institute (IMSS), catering to individuals in the formal industrial and service sectors; the Institute for Social Security and Services for State Workers (ISSSTE), serving government employees; Mexican Petroleum (PEMEX), extending coverage to workers in the national petroleum sector; and, finally, the Secretary of National Defense (SEDENA) and the Navy (SEMAR), providing coverage to members of the armed forces. Conversely, social welfare institutions encompass entities such as the Mexican Ministry of Health (SS), the federal program IMSS-*Bienestar*, and various state and municipal institutions [20].

Estimates of the population covered by these health institutions rely on diverse data sources. However, the number of beneficiaries within social security institutions often serves as a proxy to gauge the population allocated to social welfare institutions. Thus, according to INEGI, 97.8% of the population was covered by social security in 2020 [19]. Nonetheless, this estimate may not accurately reflect the demand for, or utilization of, both public and private healthcare services.

Under this fragmented and segmented organizational scheme, institutions independently plan and administer their resources to meet the demands of their designated population. In the case of obstetric care, according to the Demographic Dynamics Survey of 2018 [21], IMSS (the main social security institution in terms of population size, serving 50% of the population) provided obstetric care to 26.5% of pregnant respondents. The remaining social security institutions (ISSSTE, SEDENA, and SEMAR) collectively served 2.8% of pregnant respondents, and the Ministry of Health served 48.2%. The remainder of pregnant women received care from private providers (21.1%) and other public providers (1.3%) [21]. Services were delivered not only by obstetricians but also by trained professionals such as licensed obstetric nurses, hospital-based midwives, and traditional midwives. Prenatal care is usually administered in primary care settings, while delivery services are provided in secondary-level settings [22].

This organizational scheme could hinder the efficient delivery of obstetric services required to meet the maternal health needs of the Mexican population, especially if it is assumed that the productive efficiency of institutions is impacted by fragmentation and segmentation. These conditions impede collaboration between institutions and shape demand in a manner that results in the overutilization of a few institutions and the underutilization of others. Furthermore, given that these institutions are part of a larger social and economic context, it is anticipated that those located in regions with better economic conditions will demonstrate higher levels of efficiency. This study aimed to evaluate the technical efficiency of the Mexican public health system in the delivery of obstetric care during the period from 2012 to 2018, by economic condition at the state level.

## 2. Materials and Methods

### 2.1. Study Design and Sample

We carried out a multi-stage quantitative study of the public health institutions responsible for delivering 95% of obstetric services from 2012 to 2018. In the first stage, we estimated and compared the efficiency of each institution’s representative decision-making unit (DMU) in each state. In the second stage, given the segmentation and fragmentation of the Mexican health system, we calculated and compared the average institutional and state technical efficiency scores. In the third stage, we cross-checked the average efficiency of each state with the state GDP per capita.

### 2.2. Inclusion and Exclusion Criteria

To fulfill the objectives of this study, only federal public health institutions maintaining records of inputs necessary for obstetric services (obstetrician-gynecologists, exam rooms, delivery rooms, and operating rooms) were included. All non-federal institutions, including state and municipal organizations, as well as institutions lacking records of the aforementioned inputs during the study period were excluded. This exclusion criterion led to the removal of IMSS-*Bienestar*, which serves the population lacking social security coverage.

The final sample comprised the following institutions: SS, IMSS, ISSSTE, PEMEX, SEDENA, and SEMAR. The first three are present in all 32 states, which is the main politico-administrative division of Mexico [23]. SEDENA is present in 25 states, SEMAR in 18, and PEMEX in 16.

### 2.3. Variable of Interest and Data Source

Table 1 includes the data sources and variables of interest: the efficiency (TE) of the public health institutions in the delivery of obstetric care, and the state GDP per capita. TE was defined as the relationship between inputs (obstetrician-gynecologists, exam rooms, delivery rooms, and operating rooms) and outputs (consultations and delivery services). These outputs correspond to the minimum care required to fulfill the basic requirements established by the Mexican government [22].

The selected inputs were combined to generate the outputs, which, while not the sole resources involved in the production process, are indispensable. Additionally, these inputs were the only ones for which data were publicly available across all institutions throughout the study period.

The data used in the analysis were open access and published by the General Directorate of Health Information of the Mexican Ministry of Health. The information regarding the outputs was obtained from the databases of rendered services including outpatient services (consultations), and hospital discharges related to delivery services. The information about the inputs was extracted from the Human, Physical, Material, and Financial Resources Database.

### 2.4. Procedure and Data Analysis

In the first stage, we estimated technical efficiency using data envelopment analysis (DEA). DEA (Supplementary Materials) is a non-parametric lineal programming technique that creates an efficient production frontier using the scores of the highest performing DMUs, with 1 being the maximum possible value. Unlike other efficiency analysis techniques such as those based on statistical and parametric methodologies, DEA does not impose a pre-established form on production functions [25]. This allows for the construction of various types of production functions: one output and one input; one output and multiple inputs; multiple outputs and one input; and multiple outputs and multiple inputs. DEA also makes it possible to define the production function’s returns to scale as constant or variable [25], and to choose between an input- or output-oriented optimization analysis. Input-oriented optimization assumes a fixed level of output and aims to minimize the quantity of inputs used to obtain the established output level. Output-oriented optimization assumes sufficient available inputs and aims to maximize the quantity of outputs [10].

In this study, the function included multiple outputs and multiple inputs, and the analysis was oriented toward input minimization. An output maximization analysis was not chosen because the organization of the health system restricted the number of possible users, limiting the production growth of each institution. The function assumed constant returns to scale, meaning that DMUs with the highest input-output quotient established the efficient frontier, and DMUs with lower quotients had unused capacity that could be leveraged to increase productivity. Furthermore, the function assumed homogeneity in the quality of outputs (consultations and deliveries) across institutions.

This assumption stemmed from the fact that all institutions constituting Mexico’s healthcare system participated in the development of the Official Mexican Standard, which stipulates the types and methods of output production. This standard mandates that care for women during pregnancy, childbirth, and the postpartum period must meet certain quality criteria [22].

The analysis included 153 DMUs representing each federal institution’s affiliate at the state level.

We estimated technical efficiency using the following equation:

TEn = *min_λ_*, *x*_i_

*st*: *yi* + *Yλ* ≥ 0,

*xi* − *Xλ* ≥ 0,

*λ* ≥ 0

where TE = technical efficiency of each DMU; *λ* = weight for the DMU; *w* = cost of inputs (obstetrician-gynecologists, exam rooms, and delivery rooms); *x* = quantity of inputs; and *y* = quantity of outputs (consultations and deliveries).

The weight of each DMU was automatically computed using the Stata statistical package (version 15). This software calculates the individual efficiency of each unit by deriving a ratio between outputs and inputs, and clusters the DMUs with the highest scores to delineate the production frontier, set at a maximum value of 1. DMUs with efficiency levels falling between 0 and 0.99 are juxtaposed against those positioned on the production frontier. In the analysis, performance thresholds were established, based on the highest institutional average (0.82) and the lowest institutional average (0.22). It was determined that values exceeding 0.82 denoted DMU saturation, while values of 0.22 or below indicated a DMU with untapped operational capacity, offering opportunities to enhance efficiency. In the second stage, we identified the efficiency gap between institutions at the national and state levels. In the third stage, we graphically and statistically analyzed (using the *pwcorr* command in Stata) the relationship between economic level (measured by the state GDP per capita), and the efficiency of the state-level public health system (the average of all the institutions with a presence in the state).

To assess the temporal stability of the efficiency levels achieved by the DMUs [26], the annual variation of inputs and outputs (elements utilized in the calculation of technical efficiency) was quantified.

## 3. Results

Table 2 provides a summary of the inputs and outputs of Mexico’s public health institutions during the analysis period. The data illustrate that Institution A stood out with the largest quantity of both inputs and outputs across all categories.

**Figure 1:**
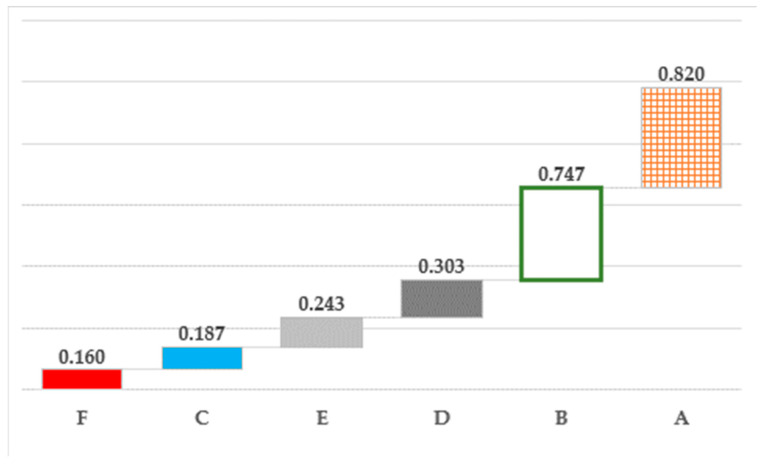
Technical efficiency of Mexico’s public health system in the delivery of obstetric care, 2012–2018.

The relationship between output and inputs at the national level, representing the average technical efficiency in the delivery of obstetric care, was 0.41 during the period from 2012 to 2018. Nevertheless, the efficiency score of its component institutions ranged widely, from 0.16 (SEMAR) to 0.82 (SS) (Figure 1). The remainder of the institutions, except for IMSS (0.747), scored far below the mean, with a collective efficiency of 0.22.

**Figure 2:**
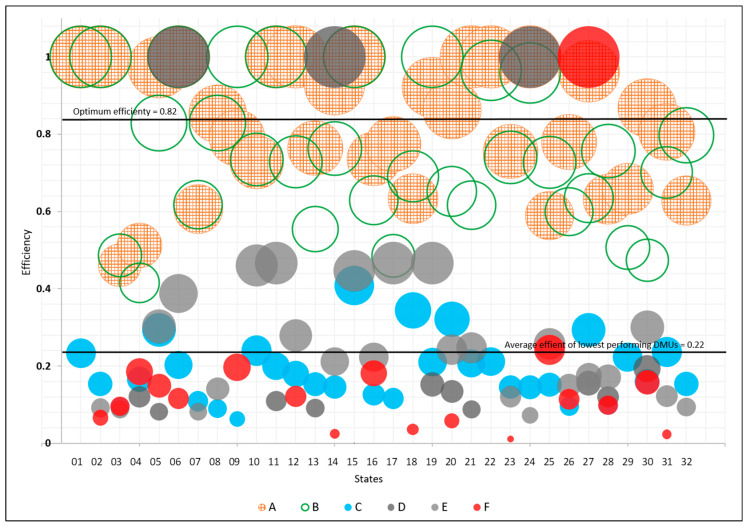
The graph depicts the efficiency of each institution across the 32 federal entities. Each institution is represented by a distinct symbol and color, with size representing efficiency score.

The technical efficiency of the institutions at the state level varied widely (Figure 2). It should be noted that the Ministry of Health’s DMUs reached the maximum level of efficiency (score = 1) in 9 out of 32 states; IMSS, in 7 states; PEMEX, in 3; and SEMAR, in 1. In contrast, ISSSTE had consistently low efficiency scores, from a minimum of 0.063 in Colima to a maximum of 0.408 in the State of Mexico. SEMAR also had very low efficiency scores (0.011 and 0.242) in 17 of its 18 states of operation. SEDENA, which operates in 25 states, scored from 0.073 to 0.467. PEMEX, with a presence in 14 states, scored highest in San Luis Potosi, Jalisco, and Chiapas. However, in its other states of operation, it scored from 0.0003 to 0.193 and was the institution with the least efficient DMU in the sample (in the State of Mexico).

The gap analysis of the institutions at the state level showed significant dispersion (Figure 2, and Table 3). Of all DMUs in the public health system (n = 153), 59.4% performed below the established optimum level (0.82), and therefore had unused operational capacity that could be leveraged to increase efficiency. In contrast, 20% of the DMUs were saturated, with efficiency scores over the established optimum level. Additionally, 75% of the DMUs pertaining to ISSSTE, PEMEX, SEDENA, and SEMAR performed below the average efficiency of the lowest performing DMUs (0.22).

**Figure 3:**
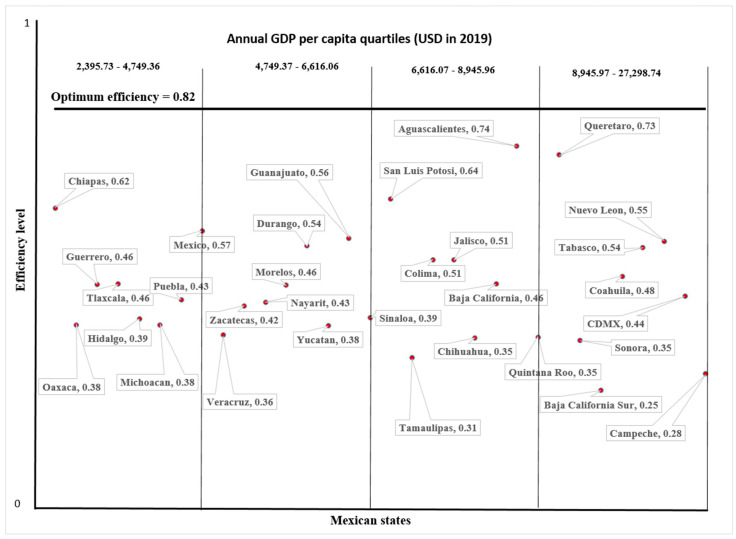
Relationship between efficiency and state GDP per capita.

The analysis of the relationship between the technical efficiency of the state-level health system and the state GDP per capita revealed no correlation between efficiency and the socioeconomic conditions of the state. Notably, distinct efficiency scores coexisted independently of GDP quartile (Figure 3). In the lower quartile (lowest GDP), efficiency ranged from 0.379 to 0.618, mirroring a similar range observed in the upper quartile (0.245–0.726). Particularly noteworthy in the upper quartile is Campeche, which has the highest GDP per capita (USD 27,298.74) and the second lowest efficiency level in the nation (0.280). Likewise, Spearman’s correlation analysis showed no statistically significant association between technical efficiency and the state GDP per capita.

Finally, Figure 3 shows that, compared to the established optimum efficiency score of 0.82, all states have unused operational capacity that could be employed to increase efficiency.

## 4. Discussion

The analysis highlights the effects of fragmentation and segmentation within Mexico’s healthcare system on the technical efficiency in the provision of obstetric services. The observed low national average technical efficiency score (0.41) does not necessarily indicate inherent inefficiency solely due to the public nature of these institutions [27,28]. Rather, efficiency scores may be attributed to current policies that assign individuals to healthcare institutions based on criteria unrelated to their health needs [29,30]. This segmentation leads to a mismatch between healthcare service supply and demand, concentrating demand in just two public institutions. These institutions bear the responsibility of providing care populations with the poorest socioeconomic conditions, including those without social security, and the majority of workers, predominantly blue-collar [20].

Mexico’s health system, when conceived of as an integral, functional unit and not as a disconnected set of public institutions, possesses the operational capacity to meet the demand for obstetric care in all states. It can therefore be inferred that the observed institutional inefficiency is due to a structural imbalance [31] attributable to the current organization of the system, which keeps the “non-beneficiary” population from accessing social security institutions.

In terms of the efficiency of individual DMUs, which represent institutions at the state level, the results demonstrate two relevant points. First, the efficiency of DMUs within the same institution varied across states. Second, efficiency varied across different states in Mexico. These results underscore that the technical efficiency of an institution can significantly vary across different regions of the country, and these variations may be associated with the size and type of the population served. Thus, there are instances where institutions possess unused service capacity, while large segments of the population paradoxically lack access to those unused public resources. This situation reflects the lack of coordination between population needs and available resources.

These inter-institutional differences—where a few institutions operate at high capacity (with high efficiency scores) while many others have unused operational capacity (resulting in low efficiency scores)—represent an opportunity to streamline service production, thereby increasing the quality of care and improving the overall efficiency of the healthcare system. To correct these imbalances, a program aimed at integrating the various health institutions was established in 2022, with the goal of resolving the longstanding issues of fragmentation and segmentation in the Mexican health system [18,32].

While it was anticipated that this study would reveal a direct correlation between efficiency and the GDP per capita at the state level, the findings indicate that efficiency is not inherently linked to this factor. However, future analyses should consider other factors that could influence the efficiency of the healthcare system, including demographic and socioeconomic characteristics of the population, macro-economic features of national and sub-national regions, population health and well-being, governance and political attributes of these regions, and health system characteristics [33].

A limitation of this study is that while this official data source (published by the General Directorate of Health Information of the Mexican Ministry of Health) allowed for a comparative analysis of efficiency of Mexican public health institutions in the delivery of obstetric care, it may not accurately reflect the functional or micro-organizational dynamics of the institutions that constitute the system. This may explain, in part, the differential performance between and within institutions. As such, we recommend that future studies account for these micro-level factors.

As previously established, the efficiency of healthcare institutions is influenced not only by organizational factors but also by environmental factors and population characteristics which shape individual and collective health. Incorporating these elements into the analysis would contribute to a better understanding of our findings and provide valuable insights for decision-making and policy design aimed at enhancing service provision. However, the data used lack the necessary level of granularity to conduct such an analysis. Subsequent studies should utilize other data sources to identify factors related to the efficiency of public health institutions in Mexico. Nevertheless, the inclusion of GDP in the present analysis, while not exhaustive, represents an initial approach to quantitatively measure the potential causes of differences in efficiency levels among public institutions providing obstetric services between 2012 and 2018 in Mexico. An additional limitation was the lack of information regarding the quality of care and the characteristics of the services rendered. However, considering the current regulatory framework in place, which establishes guidelines and standardizes obstetric care across all healthcare institutions in Mexico [22], it would be expected that significant differences in the quality and type of services provided by each institution do not exist.

This analysis did not account for the potential effects of the COVID pandemic, as the structure of the healthcare system and the inputs and outputs did not undergo significant modifications. Obstetric care remained one of the services continuously provided, even during the acute phase of the pandemic [34].

## 5. Conclusions

The results of this analysis indicate that pertaining to the public sector is not a necessary and sufficient condition to explain the problems of productive inefficiency in Mexico’s healthcare system. Despite all institutions included in the analysis belonging to the public healthcare sector, differential levels of efficiency were observed.

The efficiency issues observed in the DMUs are not inherently related to a specific institution or the state’s GDP per capita. Varied levels of efficiency were observed in DMUs belonging to the same institution but located in different states. Additionally, it was noted that states with a high GDP per capita exhibited low levels of productive efficiency in obstetric services, and conversely, states with a low GDP per capita exhibited higher levels of efficiency. Thus, it is concluded that organizational and structural factors are the primary determinants of efficiency in public institutions producing obstetric services in Mexico.

Structural factors that influence the supply and demand of healthcare services, such as system fragmentation and segmentation, result in saturation in some healthcare facilities and underutilization in others. Our findings indicate that institutions with the lowest efficiency scores are those catering to specific population segments (such as government workers, members of the national armed forces, and oil workers), leading to an excess of resources compared to the number of users requiring obstetrical care. Conversely, institutions with the highest efficiency scores are those serving the majority of the population (including formal sector workers and individuals without social security), resulting in a shortage of resources relative to the demand for care. These discrepancies between available resources and the size of the beneficiary population across institutions create disparities between outputs and inputs. Consequently, two of the institutions have very high efficiency scores while the other four score very low, culminating in a low average efficiency level within Mexico’s healthcare system.

It is expected that the integration of Mexico’s public health institutions into a single system would help optimize available resources while achieving the objectives of the healthcare system. Integration could begin with the negotiation of collaborative agreements between institutions, allowing free access to health services for individuals regardless of their insurance status. Additionally, legal reforms are required to unlink the right to use services from employment type and to structure the provision of services according to the health needs of the population.

## Figures and Tables

**Table 1:** Variables of interest and data sources.

Type of Variable	Variable	Description	Sources
**Dependent**	**Technical efficiency**	**Conceptual definition**	Estimated
		Relationship between outputs and inputs that maximizes the level of product or minimizes costs [24]	
		**Operational definition**	
		Quotient between the sum of services produced and the resources used in their production.	
**Independent**	**Inputs**	**Conceptual definition**	
		A component of production (such as land, labor, or raw materials)	
		**Operational definition**	
		Gynecologists, exam rooms, delivery rooms, and operating rooms used by Mexican Federal health institutions to produce obstetric services during the analysis period (2012–2018).	Health Resource Base. Dynamic cubes. Human, Physical, Material and Financial Resources. 2012–2018, platform (SINAIS). Available online: http://www.dgis.salud.gob.mx/contenidos/basesdedatos/bdc_recursos_gobmx.html (accessed on 11 May 2021)
		**Conceptual definition**	
	**Outputs**	The quantity of goods or services produced in a specific period.	
		**Operational definition**	
		Prenatal visits, cesareans, deliveries, and abortions produced by Mexican federal health institutions during the analysis period (2012–2018).	1. Base of Services Provided. Dynamic cubes. 2012–2018 platform (SIS). Available online: http://www.dgis.salud.gob.mx/contenidos/basesdedatos/bdc_serviciossis_gobmx.html (accessed on 11 May 2021) 2. Base of Hospital Discharges. Dynamic cubes. 2012–2018 SAEH, platform. Available online: http://www.dgis.salud.gob.mx/contenidos/basesdedatos/bdc_egresoshosp_gobmx.html (accessed on 11 May 2021)
**Contextual characteristics**	**State gross domestic product per capita**	**Conceptual definition**	
		The quotient between GDP, defined as “the standard measure of the value added created through the production of goods and services in a country during a certain period”, and the state population.	
		**Operational definition**	
		Calculated and published by The National Institute of Statistics and Geography of Mexico (INEGI).	INEGI. Per capita gross domestic product. Available online: https://cuentame.inegi.org.mx/economia/pibpc.aspx?tema=e (accessed on 11 May 2021)

**Table 2:** Outputs and inputs of Mexico’s public health institutions, 2012–2018.

	Institutions						Total
**Outputs**	A	B	C	D	E	F	
Prenatal visits	46,993,718	33,035,961	5,386,732	1,080,422	777,644	284,385	87,558,862
	53.7%	37.7%	6.2%	1.2%	0.9%	0.3%	
Deliveries	2,945,997	1,065,349	50,389	4714	25,895	4468	4,096,812
	71.9%	26.0%	1.2%	0.1%	0.6%	0.1%	
Cesarean	1,596,098	884,649	114,625	11,646	14,295	6077	2,627,390
	60.7%	33.7%	4.4%	0.4%	0.5%	0.2%	
Abortion	586,055	270,069	31,145	2043	1112	306	890,730
	65.8%	30.3%	3.5%	0.2%	0.1%	0.0%	
**Inputs**							
Gynecologists	4778	3832	1109	110	71	43	9943
	48.1%	38.5%	11.2%	1.1%	0.7%	0.4%	
Exam rooms	1041	429	223	45	84	38	1860
	56.0%	23.1%	12.0%	2.4%	4.5%	2.0%	
Delivery rooms	2794	393	150	24	44	27	3432
	81.4%	11.5%	4.4%	0.7%	1.3%	0.8%	
Cesarean sections	1887	1318	330	74	98	45	3752
	50.3%	35.1%	8.8%	2.0%	2.6%	1.2%	

**Table 3:** Technical efficiency of decision-making units (DMUs) within each state and the state-level health system in the delivery of obstetric care, Mexico, 2012–2018.

State	A	B	C	D	E	F	Average State TE
Aguascalientes	1.000	1.000	0.233				0.744
Baja California	1.000	1.000	0.154		0.092	0.067	0.463
Baja California Sur	0.461	0.486	0.094		0.088	0.096	0.245
Campeche	0.512	0.416	0.166	0.120		0.185	0.280
Mexico City	0.973	0.829	0.294	0.082	0.303	0.149	0.438
Chiapas	1.000	1.000	0.203	1.000	0.388	0.116	0.618
Chihuahua	0.606	0.618	0.109		0.082		0.354
Coahuila	0.854	0.829	0.090		0.141		0.478
Colima	0.791	1.000	0.063			0.196	0.513
Durango	0.727	0.735	0.240		0.460		0.541
Guanajuato	1.000	1.000	0.200	0.110	0.467		0.555
Guerrero	1.000	0.729	0.180		0.278	0.121	0.462
Hidalgo	0.765	0.555	0.153	0.092			0.391
Jalisco	0.928	0.763	0.146	1.000	0.211	0.025	0.512
Mexico State	1.000	1.000	0.408	0.000	0.446		0.571
Michoacán	0.736	0.629	0.127		0.223	0.181	0.379
Morelos	0.774	0.485	0.116		0.467		0.460
Nayarit	0.634	0.691	0.343			0.036	0.426
Nuevo León	0.922	1.000	0.210	0.153	0.466		0.550
Oaxaca	0.864	0.652	0.322	0.134	0.242	0.059	0.379
Puebla	1.000	0.616	0.207	0.087	0.247		0.431
Querétaro	1.000	0.966	0.212				0.726
Quintana Roo	0.756	0.740	0.145		0.121	0.011	0.355
San Luis Potosí	1.000	0.959	0.145	1.000	0.073		0.635
Sinaloa	0.590	0.727	0.152		0.258	0.242	0.394
Sonora	0.780	0.600	0.096		0.149	0.114	0.348
Tabasco	0.963	0.634	0.293	0.156	0.175	1.000	0.537
Tamaulipas	0.632	0.757	0.099	0.120	0.171	0.099	0.313
Tlaxcala	0.659	0.507	0.223				0.463
Veracruz	0.869	0.474	0.160	0.193	0.300	0.157	0.359
Yucatán	0.806	0.701	0.237		0.122	0.023	0.378
Zacatecas	0.628	0.797	0.154		0.094		0.418
Average Institutional TE	0.820	0.747	0.187	0.303	0.243	0.160	0.460

## Data Availability

The primary data were obtained from public databases, available on the Internet, as mentioned in Table 1.

## Supplementary Materials

The following supporting information can be downloaded at: https://www.mdpi.com/article/10.3390/healthcare12060653/s1. Detailed description of the performed DEA.
