# Supplementary material for: Technical Efficiency of Mexico’s Public Health System in the Delivery of Obstetric Care, during 2012–2018
Source: Healthcare (Basel). 2024 Mar 14;12(6):653. doi: 10.3390/healthcare12060653 (PMC10970456; doi:10.3390/healthcare12060653)
Supplement: Supplementary file 1 [file healthcare-12-00653-s001.zip › healthcare-2836159-supplementary.pdf]

Supplementary Materials

Detailed description of the performed DEA

I. Construction of the Database:

This information was compiled in Dynamic Cubes format, specifically utilizing the Hospital Discharge, Resources, and Services Provided Cubes (http://www.dgis.salud.gob.mx/contenidos/basesdedatos/BD_Cubos_gobmx.html). These databases were transformed through an import process from Excel format to SQL and STATA. Subsequently, a catalog of healthcare institutions was created to reconcile any discrepancies in values among the three databases.

As an illustrative example, the process followed to obtain hospital discharge data for the year 2012 is presented below. This same procedure was followed for the remaining years and databases.

1. Importation of hospital discharges for 2012 and transformation of Excel files to MS SQL

Tabla dinámica de Microsoft Office 10.0

Afección principal CIE

(Varios elementos)

Motivo

			CURACION		MEJORIA		VOLUNTARIO		PASE A OTRO HOSPITAL		DEFUNCION		OTRO MOTIVO		NO ESPECIFICADO		Total general	
Año	Entidad	Institución	Días estancia	Egresos	Días estancia	Egresos	Días estancia	Egresos	Días estancia	Egresos	Días estancia	Egresos	Días estancia	Egresos	Días estancia	Egresos	Días estancia	Egresos
⊟ 2012	⊟ Aguascalientes	SALUD	1	1	6,277	3,865	4	2	8	7	3	2	1	1			6,294	3,878
		IMSS			2,738	1,191	12	3					6	3			2,756	1,197
		ISSSTE			179	132	15	4									194	136
		Total	1	1	9,194	5,188	31	9	8	7	3	2	7	4			9,244	5,211
	⊟ Baja California	SALUD	1,477	832	8,004	4,316	22	17	25	45	1	2	6	6	1	7	9,536	5,225
		IMSS	69	42	9,139	3,925	49	27	64	46			35	22			9,356	4,062
		IMSS OPORTUNIDADES	1	1	416	182	2	2	30	25			1	1			450	211
		ISSSTE	2	1	488	260	2	2									492	263
		SEMAR	6	3	144	45											150	48
		Total	1,555	879	18,191	8,728	75	48	119	116	1	2	42	29	1	7	19,984	9,809
	⊟ Baja California Sur	SALUD	1,366	892	1,284	692	22	11	16	11			1	1			2,689	1,607
		IMSS	46	30	3,582	1,955	42	28	10	7			90	49			3,770	2,069
		ISSSTE	13	7	512	240	1	1	9	6							535	254
		SEMAR	2	2	28	10											30	12
		Total	1,427	931	5,406	2,897	65	40	35	24			91	50			7,024	3,942
	⊟ Campeche	SALUD	258	128	5,159	2,532	75	49	113	79	23	2	6	4	62	29	5,696	2,823
		IMSS	532	213	3,673	1,721	64	39	64	28			26	18			4,359	2,019
		IMSS OPORTUNIDADES	9	4	321	132	97	26	42	30			5	3			474	195
		ISSSTE	4	3	246	108	3	3	6	5							259	119
		PEMEX			642	178	17	7	7	2							666	187
		SEMAR			61	26			6	2							67	28
		Total	803	348	10,102	4,697	256	124	238	146	23	2	37	25	62	29	11,521	5,371
	⊟ Coahuila de Zaragoza	SALUD	82	52	8,907	4,923	99	74	117	80			12	8			9,217	5,137
		IMSS	89	44	15,810	7,266	172	81	150	81			134	65			16,355	7,537
		IMSS OPORTUNIDADES	12	3	1,825	676	18	8	84	55			12	5			1,951	747
		ISSSTE			760	488	10	7	10	8							780	503
		Total	183	99	27,302	13,353	299	170	361	224			158	78			28,303	13,924
	⊟ Colima	SALUD	2	2	2,927	1,745	2	1									2,931	1,748
		IMSS	25	11	2,107	1,032	8	4	16	7			20	12			2,176	1,066
		ISSSTE	2	1	211	82											213	83
		SEMAR	2	1	192	88					1	1					195	90
		Total	31	15	5,437	2,947	10	5	16	7	1	1	20	12			5,515	2,987
	⊟ Chiapas	SALUD	2,447	1,247	15,359	8,393	190	119	362	263	9	5	10	9			18,377	10,036
		IMSS	18	8	3,883	1,703	4	3	10	8			12	6			3,927	1,728
		IMSS OPORTUNIDADES	17	7	6,928	3,032	305	106	232	91			32	17			7,514	3,253
		ISSSTE	16	7	769	331	1	1	2	1							788	340
		SEMAR			60	26											60	26
		Total	2,498	1,269	26,999	13,485	500	229	606	363	9	5	54	32			30,666	15,383
	⊞ Chihuahua		1,521	718	24,255	10,665	257	108	355	191	3	2	160	63	22	6	26,573	11,753
	⊞ Distrito Federal		699	191	99,995	36,143	973	364	425	164	80	14	230	46			102,402	36,922
	⊞ Durango		120	65	11,676	6,788	77	45	84	62	17	4	16	8			11,990	6,972
	⊞ Guanajuato		925	488	44,472	23,200	424	208	533	336	58	9	83	44			46,495	24,285

Año	Entidad	DH	1DE	1EG	2DE	2EG	3DE	3EG	4DE	4EG	5DE	5EG	6DE	6EG	7DE	7EG	TGDE	TGEG
2012	Aguascalientes	SALUD	2	2	24938	19510	14	11	74	32	14	7	6	5	2	1	25050	19568
2012	Aguascalientes	IMSS	20	12	9312	4632	22	8	14	5	0	0	12	7	0	0	9380	4664
2012	Aguascalientes	ISSSTE	1	1	898	626	17	6	1	1	0	0	5	1	0	0	922	635
2012	Baja California	SALUD	8094	5447	40738	23632	46	34	59	104	1	2	11	8	8	19	48957	29246
2012	Baja California	IMSS	302	154	32540	15179	94	59	139	104	14	1	83	44	0	0	33172	15541
2012	Baja California	IMSS OPORTUNIDADES	1	1	3437	1632	63	32	142	105	0	0	20	16	0	0	3663	1786
2012	Baja California	ISSSTE	2	1	1673	882	3	3	1	1	0	0	1	1	0	0	1680	888
2012	Baja California	SEMAR	6	3	439	163	0	0	0	0	0	0	0	0	0	0	445	166
2012	Baja California Sur	SALUD	8116	5849	3675	2252	40	26	57	44	0	0	20	9	0	0	11908	8180
2012	Baja California Sur	IMSS	162	103	8722	5125	58	37	27	15	0	0	168	107	0	0	9137	5387
2012	Baja California Sur	ISSSTE	93	50	1420	787	2	2	30	11	0	0	6	2	0	0	1551	852
2012	Baja California Sur	SEMAR	4	3	92	46	0	0	0	0	0	0	0	0	0	0	96	49
2012	Campeche	SALUD	2226	1149	21264	12361	162	97	328	271	35	4	33	19	217	105	24265	14006
2012	Campeche	IMSS	1673	658	7505	3492	87	56	167	73	0	0	56	36	0	0	9488	4315
2012	Campeche	IMSS OPORTUNIDADES	33	15	2833	1005	250	108	170	82	0	0	46	18	0	0	3332	1228
2012	Campeche	ISSSTE	158	68	701	328	4	4	12	11	1	1	0	0	0	0	876	412
2012	Campeche	PEMEX	3	1	1206	325	17	7	15	5	0	0	0	0	0	0	1241	338
2012	Campeche	SEMAR	3	2	164	72	0	0	8	4	0	0	0	0	0	0	175	78
2012	Coahuila de Zaragoza	SALUD	711	527	37951	24672	280	195	383	304	21	3	58	34	1	1	39405	25736
2012	Coahuila de Zaragoza	IMSS	290	139	41768	20126	378	194	319	175	3	2	236	134	0	0	42994	20770
2012	Coahuila de Zaragoza	IMSS OPORTUNIDADES	20	7	8969	3289	91	43	284	197	0	0	41	15	0	0	9405	3551
2012	Coahuila de Zaragoza	ISSSTE	3	3	3402	1955	15	12	26	21	1	1	3	3	0	0	3450	1995
2012	Colima	SALUD	15	11	14953	10189	4	3	2	2	1	1	1	1	0	0	14976	10207
2012	Colima	IMSS	63	35	6971	4054	22	12	37	14	0	0	105	71	0	0	7198	4186
2012	Colima	ISSSTE	2	1	713	390	8	2	0	0	0	0	0	0	0	0	723	393
2012	Colima	SEMAR	214	120	346	190	0	0	1	1	1	1	0	0	0	0	562	312
2012	Chiapas	SALUD	13229	8348	76228	50249	500	301	863	685	43	14	44	36	2	2	90909	59635

2. Conversion of alphanumeric data to numeric data

Año	Entidad	DH	1DE	1EG	2DE	2EG	3DE	3EG	4DE	4EG	5DE	5EG	6DE	6EG	7DE	7EG	TGDE	TGEG
2012	1	1	2	2	24938	19510	14	11	74	32	14	7	6	5	2	1	25050	19568
2012	1	5	20	12	9312	4632	22	8	14	5	0	0	12	7	0	0	9380	4664
2012	1	6	1	1	898	626	17	6	1	1	0	0	5	1	0	0	922	635
2012	2	1	8094	5447	40738	23632	46	34	59	104	1	2	11	8	8	19	48957	29246
2012	2	5	302	154	32540	15179	94	59	139	104	14	1	83	44	0	0	33172	15541
2012	2	2	1	1	3437	1632	63	32	142	105	0	0	20	16	0	0	3663	1786
2012	2	6	2	1	1673	882	3	3	1	1	0	0	1	1	0	0	1680	888
2012	2	9	6	3	439	163	0	0	0	0	0	0	0	0	0	0	445	166
2012	3	1	8116	5849	3675	2252	40	26	57	44	0	0	20	9	0	0	11908	8180
2012	3	5	162	103	8722	5125	58	37	27	15	0	0	168	107	0	0	9137	5387
2012	3	6	93	50	1420	787	2	2	30	11	0	0	6	2	0	0	1551	852
2012	3	9	4	3	92	46	0	0	0	0	0	0	0	0	0	0	96	49
2012	4	1	2226	1149	21264	12361	162	97	328	271	35	4	33	19	217	105	24265	14006
2012	4	5	1673	658	7505	3492	87	56	167	73	0	0	56	36	0	0	9488	4315
2012	4	2	33	15	2833	1005	250	108	170	82	0	0	46	18	0	0	3332	1228
2012	4	6	158	68	701	328	4	4	12	11	1	1	0	0	0	0	876	412
2012	4	7	3	1	1206	325	17	7	15	5	0	0	0	0	0	0	1241	338
2012	4	9	3	2	164	72	0	0	8	4	0	0	0	0	0	0	175	78
2012	5	1	711	527	37951	24672	280	195	383	304	21	3	58	34	1	1	39405	25736
2012	5	5	290	139	41768	20126	378	194	319	175	3	2	236	134	0	0	42994	20770
2012	5	2	20	7	8969	3289	91	43	284	197	0	0	41	15	0	0	9405	3551
2012	5	6	3	3	3402	1955	15	12	26	21	1	1	3	3	0	0	3450	1995
2012	6	1	15	11	14953	10189	4	3	2	2	1	1	1	1	0	0	14976	10207
2012	6	5	63	35	6971	4054	22	12	37	14	0	0	105	71	0	0	7198	4186
2012	6	6	2	1	713	390	8	2	0	0	0	0	0	0	0	0	723	393
2012	6	9	214	120	346	190	0	0	1	1	1	1	0	0	0	0	562	312
2012	7	1	13229	8348	76228	50249	500	301	863	685	43	14	44	36	2	2	90909	59635
2012	7	5	62	32	10916	5460	20	12	28	25	1	1	24	14	0	0	11051	5544
2012	7	2	65	24	39119	19361	864	337	556	283	0	0	122	74	0	0	40726	20079
2012	7	6	133	48	3609	1554	14	6	13	5	0	0	9	5	0	0	3778	1618
[illegible]	[illegible]	[illegible]	[illegible]	[illegible]	[illegible]	[illegible]	[illegible]	[illegible]	[illegible]	[illegible]	[illegible]	[illegible]	[illegible]	[illegible]	[illegible]	[illegible]	[illegible]	[illegible]

3. Importation into STATA

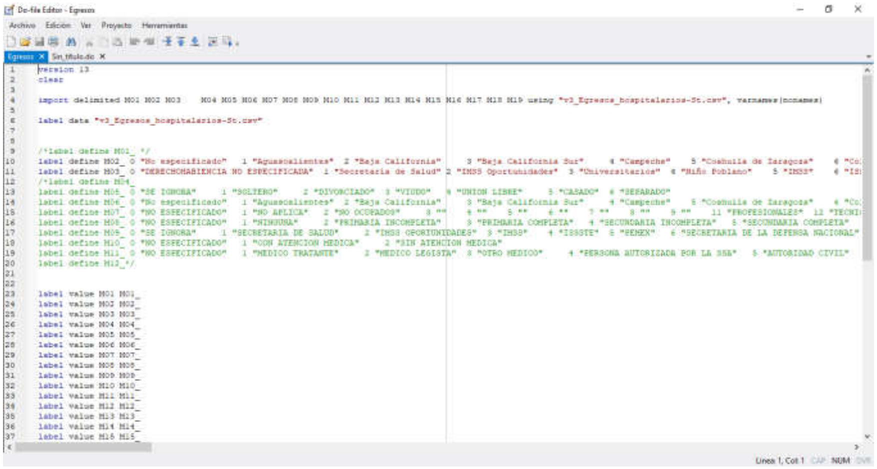
Do-file Editor - Egresos
Archivo Edición Ver Proyecto Herramientas
Egresos
Sin_titulo.do
version 13
clear
import delimited M01 M02 M03 M04 M05 M06 M07 M08 M09 M10 M11 M12 M13 M14 M15 M16 M17 M18 M19 using "v3_Egresos_hospitalarios-St.csv", varnames(nonames)
label data "v3_Egresos_hospitalarios-St.csv"
/*label define M01_ */
label define M02_ 0 "No especificado" 1 "Aguascalientes" 2 "Baja California" 3 "Baja California Sur" 4 "Campeche" 5 "Coahuila de Zaragoza" 6 "Co
label define M03_ 0 "DERECHOHABIENCIA NO ESPECIFICADA" 1 "Secretaria de Salud" 2 "IMSS Oportunidades" 3 "Universitarios" 4 "Niño Poblano" 5 "IMSS" 6 "IS
/*label define M04_
label define M05_ 0 "SE IGNORA" 1 "SOLTERO" 2 "DIVORCIADO" 3 "VIUDO" 4 "UNION LIBRE" 5 "CASADO" 6 "SEPARADO"
label define M06_ 0 "No especificado" 1 "Aguascalientes" 2 "Baja California" 3 "Baja California Sur" 4 "Campeche" 5 "Coahuila de Zaragoza" 6 "Co
label define M07_ 0 "NO ESPECIFICADO" 1 "NO APLICA" 2 "NO OCUPADOS" 3 "" 4 "" 5 "" 6 "" 7 "" 8 "" 9 "" 11 "PROFESIONALES" 12 "TECNI
label define M08_ 0 "NO ESPECIFICADO" 1 "NINGUNA" 2 "PRIMARIA INCOMPLETA" 3 "PRIMARIA COMPLETA" 4 "SECUNDARIA INCOMPLETA" 5 "SECUNDARIA COMPLETA"
label define M09_ 0 "SE IGNORA" 1 "SECRETARIA DE SALUD" 2 "IMSS OPORTUNIDADES" 3 "IMSS" 4 "ISSSTE" 5 "PEMEX" 6 "SECRETARIA DE LA DEFENSA NACIONAL"
label define M10_ 0 "NO ESPECIFICADO" 1 "CON ATENCION MEDICA" 2 "SIN ATENCION MEDICA"
label define M11_ 0 "NO ESPECIFICADO" 1 "MEDICO TRATANTE" 2 "MEDICO LEGISTA" 3 "OTRO MEDICO" 4 "PERSONA AUTORIZADA POR LA SSA" 5 "AUTORIDAD CIVIL"
label define M12_*/
label value M01 M01_
label value M02 M02_
label value M03 M03_
label value M04 M04_
label value M05 M05_
label value M06 M06_
label value M07 M07_
label value M08 M08_
label value M09 M09_
label value M10 M10_
label value M11 M11_
label value M12 M12_
label value M13 M13_
label value M14 M14_
label value M15 M15_
Línea 1, Col 1 CAP NUM OVR

4. Data output in STATA

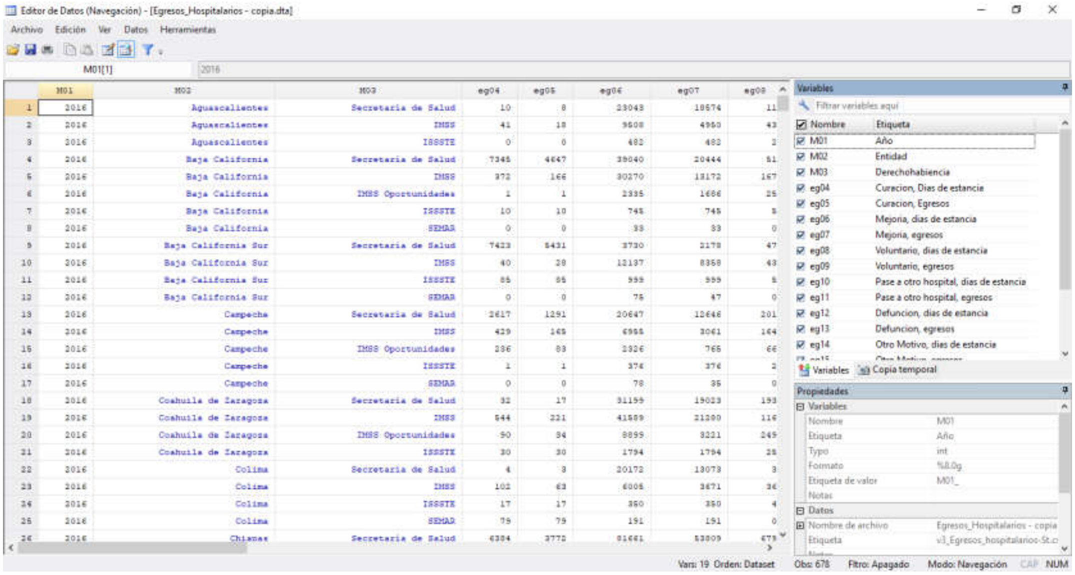

Information obtained from each database:

The data corresponding to the inputs (physicians, examination rooms, and delivery rooms) were extracted from the Dynamic Cubes Resources; consultations were extracted from the Services Provided Cube; and deliveries and cesarean sections were obtained from the Hospital Discharge Cube.

II. Efficiency Analysis (DEA):

To conduct the efficiency analysis using Data Envelopment Analysis (DEA), the following was required:

1) An identifying variable for the evaluated organizations, referred to as DMU, was established. In this study, the DMUs corresponded to the state division of each federal health institution. Therefore, the identifier for each DMU consisted of three digits. The first digit corresponded to the institution, and the other two identified the state. The institutions were coded as follows: 1) Ministry of Health; 2) IMSS; 3) ISSSTE; 4) PEMEX; 5) SEDENA; and 6) SEMAR, while the states were numbered consecutively in alphabetical order. Thus, DMU 101 corresponds to the Ministry of Health in the state of Aguascalientes, DMU 202 represents IMSS in the state of Baja California, and so on.
2) Rename the input and output variables as "ivars" and "ovars," respectively. Therefore, in this study, the variables corresponding to inputs were named as "i_doctor," "i_examroom," "i_deliveryroom," and

"i_operatingroom," while the variables corresponding to outputs were named as "o_visit," "o_birth," and "o_cesarean.

3) Install the package "st0193" from http://www.stata-journal.com/software/sj10-2.
